# Complete Genome Sequence of JII-1961, a Bovine *Mycobacterium avium* subsp. *paratuberculosis* Field Isolate from Germany

**DOI:** 10.1128/genomeA.00870-17

**Published:** 2017-08-24

**Authors:** Petra Möbius, Gabriele Nordsiek, Martin Hölzer, Michael Jarek, Manja Marz, Heike Köhler

**Affiliations:** a Institute of Molecular Pathogenesis, Friedrich-Loeffler-Institut (Federal Research Institute for Animal Health), Jena, Germany; b Department of Genome Analysis, Helmholtz Centre for Infection Research, Braunschweig, Germany; c RNA Bioinformatics and High-Throughput Analysis, Faculty of Mathematics and Computer Science, Friedrich-Schiller-Universität, Jena, Germany

## Abstract

*Mycobacterium avium* subsp. *paratuberculosis* causes Johne’s disease in ruminants and was also detected in nonruminant species, including human beings, and in milk products. We announce here the 4.829-Mb complete genome sequence of the cattle-type strain JII-1961 from Germany, which is very similar to cattle-type strains recovered from different continents.

## GENOME ANNOUNCEMENT

*Mycobacterium avium* subsp. *paratuberculosis* is an obligate pathogen in domestic and wild ruminants. It causes a chronic progressive granulomatous enteritis designated Johne’s disease (JD), or paratuberculosis, which is distributed worldwide (1). Furthermore, *M. avium* subsp. *paratuberculosis* can opportunistically induce infections in the intestine and other tissues of monogastric host species with (2) and without (3) clinical signs. *M. avium* subsp. *paratuberculosis* also has been detected in humans; its role in Crohn’s disease is under continuing critical discussion (4). Based on different phenotypes, genotypes, and host associations, *M. avium* subsp. *paratuberculosis* was divided into two main groups: the cattle type and the sheep type (5). Until now, five complete cattle-type genome sequences from three different regions of the world have been published, with four bovine derived and one human derived: *M. avium* subsp. *paratuberculosis* and *M. avium* subsp. *paratuberculosis* K10 from United States (6, 7), *M. avium* subsp. *paratuberculosis* E1 and *M. avium* subsp. *paratuberculosis* E93 from Egypt (8), and MAP/TANUVAS/TN/India/2008 from India (NCBI GenBank accession number CP015495). A genome sequence from a cattle-type strain originating from Europe has not yet been formally published.

This paper announces the complete genome sequence of the cattle-type field strain JII-1961 from Germany. This strain was isolated in 2003 from an ileocecal lymph node of a clinically diseased cow and was characterized by multitarget genotyping (9). Furthermore, JII-1961 was used as an inoculation strain for a new defined animal model for JD in goats focusing on the period of latent infection (10).

For sequencing, JII-1961 had been grown for 6 weeks in Herrold’s egg yolk medium supplemented with mycobactin J. High-quality bacterial genomic DNA was extracted using the cetyltrimethylammonium bromide method (11). Libraries were prepared with a paired-end DNA sample prep kit (Illumina, San Diego, CA, USA), assessed by a 2100 Bioanalyzer (Agilent Technologies) and sequenced on the Illumina GAIIx sequencing platform at the Helmholtz Centre for Infection Research in Braunschweig by running 36 cycles in both directions. Image analysis and base calling were performed using the Genome Analyzer Pipeline 1.4 and CASAVA software 1.7. Altogether, 5,154,552 paired-end reads with a length of 35 bp were assembled using Velvet 0.7.55-1 (12), using *M. avium* subsp. *paratuberculosis* K10 as a reference (GenBank accession no. AE016958). The remaining sequence gaps were closed by PCR and Sanger sequencing (ABI Prism 3730xl DNA analyzer). Further analysis and assembly of these additional data were done by Gap4 (Staden Package 1.6). For genome annotation, the NCBI Prokaryotic Genome Annotation Pipeline was used. In addition, noncoding RNA (ncRNA), rRNA, and tRNA genes were detected by a homology search of Rfam (version 11.0) families using the GORAP pipeline comprising Infernal (version 1.1), RNAmmer (version 1.2), and tRNAscan-SE (version 1.3.1), as described by Möbius et al. (13).

Finally, the complete chromosomal sequence of JII-1961 comprises 4,829,728 bp. The average G+C content is 69.3 mol%. NCBI genome annotation identified and predicted 4,325 protein coding sequences (CDSs), 46 tRNAs, 3 rRNAs, and 3 other ncRNAs. Using different approaches for ncRNA annotation, 46 tRNAs and 3 rRNAs were also detected, but in addition, one transfer-messenger RNA (tmRNA), one RNase P, one bacterial small signal recognition particle (SRP), 15 riboswitches, and 14 other ncRNAs were detected (altogether, 81 noncoding RNAs [13]).

### Accession number(s).

The whole-genome sequence of *Mycobacterium avium* subsp. *paratuberculosis* strain JII-1961 has been deposited at DDBJ/EMBL/GenBank under the accession no. CP022105. The version described in this paper is the first version, CP022105.1.
